# Effect of *Eurycoma longifolia* Stem Extract on Uric Acid Excretion in Hyperuricemia Mice

**DOI:** 10.3389/fphar.2019.01464

**Published:** 2019-12-10

**Authors:** Ruixia Bao, Mengyang Liu, Dan Wang, Shaoshi Wen, Haiyang Yu, Yi Zhong, Zheng Li, Yi Zhang, Tao Wang

**Affiliations:** ^1^Institute of Traditional Chinese Medicine, Tianjin University of Traditional Chinese Medicine, Tianjin, China; ^2^Tianjin State Key Laboratory of Modern Chinese Medicine, Tianjin University of Traditional Chinese Medicine, Tianjin, China; ^3^Key Laboratory of Pharmacology of Traditional Chinese Medical Formulae (Tianjin University of Traditional Chinese Medicine), Ministry of Education, Tianjin, China; ^4^Herb Research Centre, Global Education Network Sdn.Bhd., Puchong, Malaysia

**Keywords:** *Eurycoma longifolia*, eurycomanol, hyperuricemia, uric acid excretion, urate reabsorption transporter

## Abstract

**Background:** *Eurycoma longifolia* is a tropical medicinal plant belonging to Simaroubaceae distributed in South East Asia. The stems are traditionally used for the treatment of sexual insufficiency, fever, hypertension, and malaria. Furthermore, it has antidiabetic and anticancer activities. Recently, it has been reported to reduce uric acid, but the mechanism is unclear.

**Hypothesis/Purpose:** The aim of this study is to explore the effect and mechanism of *E. longifolia* stem 70% ethanol extract (EL) and its active compounds on uric acid excretion.

**Study Design and Methods:** Potassium oxonate (PO) induced hyperuricemia rats model and adenine-PO induced hyperuricemia mice model were used to evaluate the effects of EL. Ultraperformance liquid chromatography was used to determine the levels of plasma or serum uric acid and creatinine. Hematoxylin-eosin staining was applied to observe kidney pathological changes, and western blot was applied to detect protein expression levels of uric acid transporters. Effects of constituents on urate uptake were tested in hURAT1-expressing HEK293T cells.

**Results:** EL significantly reduced serum and plasma uric acid levels at dosages of 100, 200, and 400 mg/kg in hyperuricemia rats and mice, increased the clearance rate of uric acid and creatinine, and improved the renal pathological injury. The protein expression levels of urate reabsorption transporter 1 (URAT1) and glucose transporter 9 were down-regulated, while sodium-dependent phosphate transporter 1 and ATP-binding cassette transporter G2 were up-regulated in the kidney after EL treatment. The quassinoids isolated from EL showed inhibitory effects on urate uptake in hURAT1-expressing HEK293T cells, and the effect of eurycomanol was further confirmed *in vivo*.

**Conclusion:** Our findings revealed that EL significantly reduced blood uric acid levels, prevented pathological changes of kidney in PO induced hyperuricemia animal model, and improved renal urate transports. We partly clarified the mechanism was related to suppressing effect of URAT1 by quassinoid in EL. This study is the first to demonstrate that EL plays a role in hyperuricemia by promoting renal uric acid excretion.

## Introduction

Hyperuricemia defined as plasma uric acid level higher than 420 µmol/L in males and 350 µmol/L in females (Johnson et al., 2018) is the major risk factor of gout and is highly related to fatty liver (Sandra et al., 2019), diabetes (Dehghan et al., 2008), hypertension (Wang et al., 2014), chronic kidney damage (Zoppini et al., 2012), and cardiovascular diseases (Martinez-Quintana et al., 2016). Hyperuricemia is more prevalent in Asia regions (Smith and March, 2015). National cross-sectional survey showed that the prevalence of hyperuricemia was 8.4% among Chinese adults in 2009–2010 (Liu et al., 2014), and meta-analysis indicated the prevalence of hyperuricemia was 13.3% in mainland of China from 2000 to 2014 (Liu, R. et al., 2015).

Hyperuricemia is caused by excessive production or insufficient excretion of uric acid (Mandal and Mount, 2015; Maiuolo et al., 2016). Uric acid is the final product of purine catabolism due to the lack of uricase in human. Xanthine oxidase (XOD) in the liver catalyzes the oxidation of hypoxanthine and xanthine to uric acid (Haidari et al., 2008). Renal excretion and intestinal excretion are the two ways for human to eliminate uric acid to maintain blood uric acid level. Uric acid handling in the kidney through four processes, including glomerular filtration, tubular reabsorption, secretion, and post-secretory reabsorption. In renal excretion way, urate transporters play an important role on uric acid reabsorption and secretion (Lipkowitz, 2012). The urate reabsorption transporter 1 (URAT1) and glucose transporter 9 (GLUT9) are located at the apical and basolateral membranes of the proximal tubular cells, respectively, which are responsible for reabsorption of uric acid in the kidney (Ichida et al., 2004; Dinour et al., 2010). Moreover, it was reported that renal organic anion transporter 1-3 (OAT1-3) located in the basolateral membrane of the kidney, sodium-dependent phosphate transporter 1 (NPT1), NPT4, and ATP-binding cassette transporter G2 (ABCG2) located in the apical membrane of the kidney were involved in renal uric acid excretion (Atsushi et al., 2002). Accordingly, reducing uric acid production or promoting uric acid excretion may be the useful therapeutic approached to hyperuricemia.

Currently, allopurinol and febuxostat (Pacher et al., 2006) are used as the first-line drug for urate-lowering, reducing the serum uric acid levels through inhibition of XOD activity. However, some patients are intolerant to allopurinol or cannot achieve the target serum uric acid level by taking recommended dose of allopurinol (Keith and Gilliland, 2011). In addition, some clinic research reported that febuxostat showed an increased risk of cardiovascular death (Becker et al., 2010). Therefore, XOD inhibitors treatment does not sufficient enough to meet clinical needs. Uricosuric agents, such as benzbromarone, interacts with multiple renal transporters to elevate uric acid excretion and is used to improve hyperuricemia (Reinders et al., 2009). However, long-term use of uricosuric agents has been reported to cause serious hepatotoxicity (Shin et al., 2011) and increase the incidence of renal urate stones. Uricosuric agents are not first choice for gout or hyperuricemia patient (Stamp et al., 2007; Strilchuk et al., 2019). Although high selective URAT1 inhibitor, such as lesinurad and verinurad, is developed as a new therapeutic agent of hyperuricemia, the safety and true effect of treat-to-target trials are still not enough (Tausche et al., 2017).

*Eurycoma longifolia* Jack is a medicinal plant distributed in southeast Asia (Rehman et al., 2016) used as a traditional medicine to treat sexual dysfunctions (Bhat and Karim, 2010). Recently, it has been reported to reduce uric acid by inhibition of XOD activity (Lianget al., 2018), but its inhibitory activity was relatively weak that could not fully clarify the urate-lowering effect *in vivo*. In this study, we performed a series of experiments to determine the anti-hyperuricemic mechanism of *E. longifolia* in regulating uric acid excretion.

## Materials and Methods

### Materials

The stems of *E. longifolia* were collected from Titi, Jelebu District, Kuala Klawang, Malaysia (2°59’58.3”N 102°04’49.7”E), and identified by Dr. Wang Tao (Institute of Traditional Chinese Medicine, Tianjin University of Traditional Chinese Medicine). Voucher specimen was deposited at the Institute of Traditional Chinese Medicine of Tianjin University of Traditional Chinese Medicine (Voucher Number: TUTCM-17-0153).

Seventy percent ethanol extract of stem from *E. longifolia* (EL) and eurycomanol, provided by the Chinese Medicine Chemistry Laboratory of Tianjin University of Traditional Chinese Medicine, stored at 25°C. The compound, eurycomanone, was purchased from Yuanye Biotechnology Co., Ltd. Shanghai, China, and its purity [high-performance liquid chromatography (HPLC) ≥ 95%].

### Animals

Sprague-Dawley (SD) rats SPF grade, 8 weeks old, were purchased from HFK Bioscience Co., Ltd. Beijing, China. Male Kunming strain mice of SPF grade, weighing 18–22 g, were purchased from Beijing Vital River Laboratory Animal Technology Co., Ltd. All animals were allowed to have a standard diet and drink and housed in experimental conditions at 25 ± 2°C, humidity 60 ± 5% with a fixed 12 h artificial light period. They were allowed at least 7 days to adapt to their living environment before the experiments. All of animal experiments designs were approved by Science and Technological Committee and the Animal Use and Care Committee of TJUTCM (No. 201610007).

### HPLC Analysis of EL

Eurycomanone is a quassinoid isolated from stems of *E. longifolia* and is used as quality control marker. HPLC-diode array detection analysis of the eurycomanone content in EL was carried out using an Agilent 1260 Infinity II system (Agilent Technology, Santa Clara, CA) with Eclipse Plus C18 column (Agilent Technologies, USA, 4.6 × 250 mm, 5 µm), and the mobile phases water (A) and acetonitrile (B) were utilized in a gradient mode (0–18 min: 91%A, 9%B; 19–35 min: 5%A, 95%B) at ambient temperature (Li, 2017).

### Potassium Oxonate Induced Acute Hyperuricemia Rats and Mice

Potassium oxonate (PO) (Sigma-Aldrich Co., MO, USA), a urate oxidase inhibitor, was applied to induce acute hyperuricemia. Male SD rats were randomly allocated into the following six groups (n = 10): (1) normal control group, (2) PO induced hyperuricemia group, (3) PO+100 mg/kg probenecid (Sigma-Aldrich Co., MO, USA) group, (4) PO+100 mg/kg EL group, (5) PO+200 mg/kg EL group, and (6) PO+400 mg/kg EL group. Animals fasted for 24 h and allowed to drink waters freely before the experiment. PO, probenecid, and EL were suspended in 5% acacia solution and administered at 10 ml/kg. Rats were intragastrically administrated with PO (200 mg/kg) after 1 h of the treatment of EL (100, 200, 400 mg/kg) or probenecid (100 mg/kg). The control rats received 5% acacia solution as a vehicle. The blank blood was collected from infraorbital venous plexus before administration of each group of rats, and then blood samples were collected at 500 ml for 1, 2, 4, and 6 h after PO administration. The blood was allowed to clot for approximately 1 h at room temperature and then centrifuged at 4°C for 10 min at 5,000*g* to separate the serum. The serum was then stored at −20°C until analyzed.

PO-induced acute hyperuricemia mice were used for eurycomanol activity screening. PO (300 mg/kg), benzbromarone (50 mg/kg), and eurycomanol (20 mg/kg) were suspended in 5% acacia solution and administered at 20 ml/kg. Mice were intragastrically administrated with PO (300 mg/kg) 1 h after the treatment of eurycomanol and benzbromarone. The blank blood was collected before administration, and then blood samples were collected at 1, 2, and 4 h after PO administration. The plasma was then stored at −20°C until analyzed.

Ultraperformance liquid chromatography (UPLC) was used to determine serum and plasma uric acid levels (Wen et al., 2019). Perchloric acid (0.3 M) 270 µl was added to 30 µl serum or plasma sample, vortex mixing and placed in ice-water bath for 30 min, and then centrifuged at 10,000*g* for 10 min at 4°C to obtain supernatants. Supernatant (200 µl) was neutralized with 50 µl Na_2_HPO_4_ solution (0.8 M) and underwent further centrifugation at 10,000*g* for 10 min at 4°C, and then the supernatant was stocked for UPLC analysis of uric acid level. UPLC analysis conditions are as follows: Waters ACQUITY UPLC system H Class (Waters Co. Ltd. USA) with a quaternary solvent manager was used to determine serum and plasma uric acid levels. Detector: photo-diode array, column: ACQUITY UPLC BEH Amide (1.7 µm, 2.1 × 50 mm), detect wavelength: 285 nm, mobile phase: 0.1% acetic acid water solution/acetonitrile = 10/90, v/v, flow rate:0.3 ml/min, the column was maintained at 30°C.

### Adenine and PO Induced Hyperuricemia Mice

In order to further verify the *in vivo* activity of EL, mice were selected as experimental subjects. Hyperuricemia animal models in mice were established by oral administration of PO and adenine (Zhang et al., 2018) (Sigma-Aldrich Co., MO, USA). Male Kunming mice were randomized into several groups (n = 12): normal control, hyperuricemia control, benzbromarone control (50 mg/kg), and EL groups (100, 200 and 400 mg/kg respectively). Benzbromarone and EL samples were suspended in 5% acacia and orally administrated with a volume of 20 ml/kg. One hour later, mice were intragastric administration of adenine (75 mg/kg/day, volume 20 ml/kg) and PO (200 mg/kg/day, volume 20 ml/kg). Mice of normal and hyperuricemia control were only administered orally the same volume of 5% acacia solution. The treatment was applied once a day in the morning for 21 consecutive days. Blood samples were collected 1 h after final administration on the 7th day, 14th day and 21th day for biochemical assays. At the end of administration, 2 and 24 h urine samples were collected with metabolic cages, and kidney tissues were quickly and carefully separated on ice-plate. The single kidney tissue was fixed at room temperature in formalin for renal histological analysis, and another kidney was stored at −80°C for the analysis of western blot. Plasma and urine uric acid levels were determined as previously described. Plasma and urine creatinine levels were determined using creatinine kit (Biosino Bio-Technology Co., Ltd.,Beijing, China) according to the manufacturers’ instructions. Clearance of uric acid (Cur) and creatinine (Ccr) were calculated by methods reported in the literature (Perez-Ruiz et al., 2002) shown as following:

$$\begin{array}{c} \text{Cur}\;=\;\text{Uv}\;\times \;\text{Uur/Sur};\\ \text{Ccr}\;=\;\text{Uv}\;\times \;\text{Ucr/Scr}; \end{array}$$

Sur, plasma uric acid level; Scr, plasma creatinine level; Uur, urinary uric acid level; Uv, urine volume.

### Histopathology of Renal Tissues

The removed kidney tissues were fixed with 4% paraformaldehyde in PBS and embedded in paraffin for histological analysis. The 5 µm-thick paraffin sections were stained with hematoxylin and eosin (H&E), and the pathological morphology of kidney tissue was photographed with Axio Imager D2 (Zeiss, Oberkochen, Germany).

### Western Blot Analysis

Kidney tissue blocks were homogenized with 10-folds of RIPA Lysis Buffer (adding 1 mmol/L PMSF; phosphatase inhibitor) under the ice bath condition, and the mixtures were centrifuged at 12,000*g* for 10 min. The supernatants were collected and the total protein concentration was determined by Cytation 5 (BioTek, Winooski, VT, USA). Samples (70 µg) were separated by 10% SDS-polyacrylamide gel electrophoresis and transferred electrophoretically onto PVDF membrane (Merck Millipore, Bedford, MA). Non-specific binding sites on the membranes were blocked with QuickBlock™ Western blocking solution (Beyotime Institute of Biotechnology, Nanjing, China). Afterwards, they were incubated overnight individually at 4°C with anti-URAT1 (ProteinTech Group. Chicago, USA), anti-GLUT9 (Millipore Co. Ltd. Bedford, MA, USA), anti-ABCG2 (Abcam plc. Cambridge, MA, USA), anti-OAT1 (Abcam plc. Cambridge, MA, USA), anti-NPT1 (ProteinTech Group. Chicago, USA), and β-actin (Abcam plc. Cambridge, MA, USA) antibodies diluted in Tris Buffered Saline with Tween 20 (TBST, Beijing Solarbio Science & Technology Co. Ltd., Beijing, China). Then the blots were washed three times with TBST and incubated with the secondary antibodies conjugated with horseradish peroxidase at room temperature for 1 h. Subsequently, blots were washed three times with TBST and then mixed with Enhanced Chemiluminescence (Millipore Co., Ltd. MA, USA). After that, the protein bands were visualized with ChemiDoc MP Imaging System (Bio-Rad, Hercules, CA, USA). The band intensities were quantified using Image J analysis software.

### RT-PCR Analysis

RNA isolation, cDNA synthesis, and real-time PCR analysis were performed as described previously (Liu, M. et al., 2015). The primer sequences used for real-time PCR were shown in **Table 1**. Results were presented as levels of expression relative to those of controls after normalization to β-actin using the 2^−△△CT^ methods. Analysis was carried out in triplicates.

**Table 1:** Sequences of the primers for real time RT-PCR analysis.

	Forward	Reverse
URAT1	TCTCCACGTTGTGCTGGTTC	GGATGTCCACGACACCAATGA
GAPDH	GGAGCGAGATCCCTCCAAAAT	GGCTGTTGTCATACTTCTCATGG

Then RT-PCR was conducted using the SYBR Green QuantiTect RT-PCR kit (Thermo Fisher Sci. Inc., St. Austin, TX, USA) through LightCycler 96 qPCR system (Roche, Basel, Switzerland). The glyceraldehyde 3-phosphate dehydrogenase (GAPDH) acted as a control for total mRNA amount. The results were detected using the 2−△△CT method.

### Establishment of hURAT1-Expressing HEK293T Cells

HEK-293T cells were cultured in Dulbecco’s modified Eagle’s medium (DMEM, Thermo SCIENTIFIC, Rockford, USA) supplemented with 10% fetal bovine serum (Thermo SCIENTIFIC, Rockford, USA) and antibiotics (Sigma-Aldrich Co., MO, USA) at 37°C in 5% CO_2_. To establish hURAT1-expressing HEK293T cells, the cDNA of hURAT1 (GenBank accession number AB071863) was subcloned into A pHB-CMV-MCS-EF1-ZSgreen-puro (Hanbio Biotechnology Co., Ltd. Shanghai, China) using restriction enzymes EcoRI (Thermo Fisher Scientific, USA). HEK293T cells were transfected with a hURAT1 expression vector by using Lipofiter™ according to the manufacturer’s protocols (Hanbio Biotechnology Co., Ltd. Shanghai, China). An empty pHB-CMV-MCS-EF1-ZSgreen-puro vector was transfected into the HEK293T cells as a control. After 24 h of transfection, the virus infection solution was discarded and replaced with fresh complete medium. Screening of cells successfully transfected with virus by treatment of virus-transfected cells with puromycin. The hURAT1-expressing HEK293T cells were used for a urate uptake experiment.

### Transporter Activity Assays in hURAT1-Expressing HEK293T Cells

The hURAT1-expressing HEK293T cells were seeded in six-well plates at a density of 1.5×10^5^ per well in DMEM containing 10% fetal bovine serum (Biological Industries Ltd. Kibbutz Beit Haemek, Israel). After 48 h incubation at 37°C in a humidified 5% CO_2_ incubator, cell culture medium was replaced with 1 ml/well chloride-free Hanks’ balanced salt solution (Cl^–^free HBSS), and then incubated for 10 min. After 10 min incubation, the cells were incubated in Cl^−^-free HBSS containing 100 µmol/L urate (Sigma-Aldrich Co., MO, USA) with constituents (50 µmol/L) at 37°C for 30 min. RDEA3170 (1 µmol/L) (Target Molecule Co., Boston, USA), a URAT1 inhibitor, was used as positive control. The urate uptake was stopped by removing the incubation buffer and adding 500 µl of ice-cold HBSS (Cl^–^free HBSS) solution. The cells were lysed using 0.4 ml of 0.1 mmol/L NaOH. The urate contents of the cells were determined by the previously described UPLC method.

### Statistical Analysis

Data are expressed as the mean ± S.E.M. Significant differences between means were evaluated by one-way analysis of variance (ANOVA) using SPSS 20.0 statistical software (version 20, SPSS; IBM, Armonk, NY, USA). LSD and Dunnett’s test were used for *post hoc* evaluations, and *P* < 0.05 was considered to represent a statistically significant difference.

## Results

### Eurycomanone Content Analysis in EL

**Figure 1:**
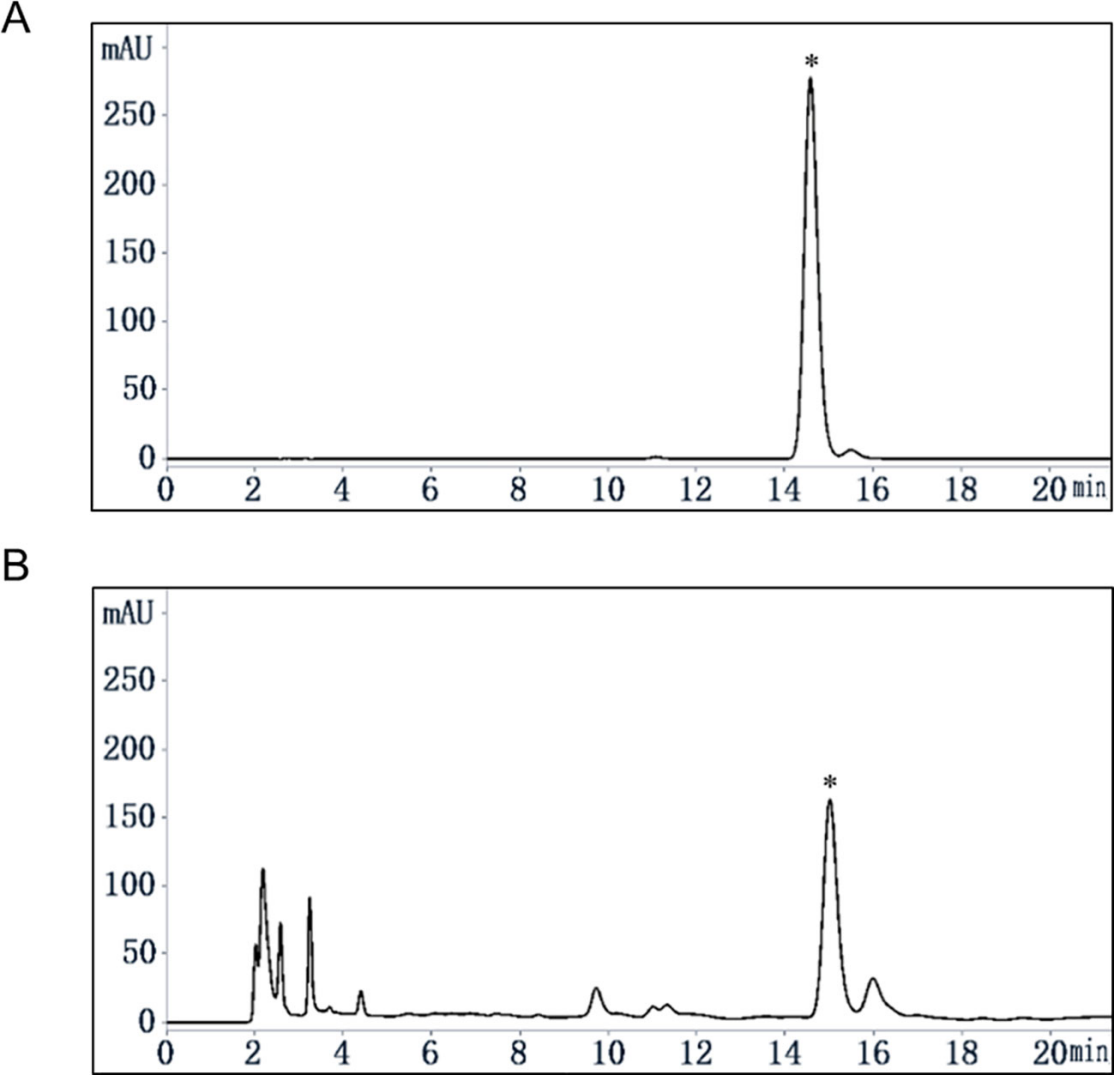
Content analysis of eurycomanone in EL by HPLC. **(A)** eurycomanone (0.4 mg/ml) standard HPLC chromatogram. **(B)** EL (1 mg/ml) HPLC chromatogram; *: eurycomanone.

Quassinoids were reported as major compounds in EL (Miyake et al., 2009), and as a further phytochemical study, our group reported 16 kinds of compounds including phenols and quassinoids from EL (Ruan et al., 2019) and the structures were identified by various spectral techniques and chemical reactions. Determination of the content of eurycomanone in EL by HPLC method reported previously (Li, 2017). The calibration curve showed a good linear relationship over a range of 0.04 to 0.40 mg/ml (Y = 15061×X-37.142, R² = 0.9998). The HPLC chromatograms were shown in **Figure 1**, and the relative content of eurycomanone in EL was 1.17%.

### EL Decreased Serum Uric Acid Levels in PO Induced Acute Hyperuricemia Rats

**Figure 2:**
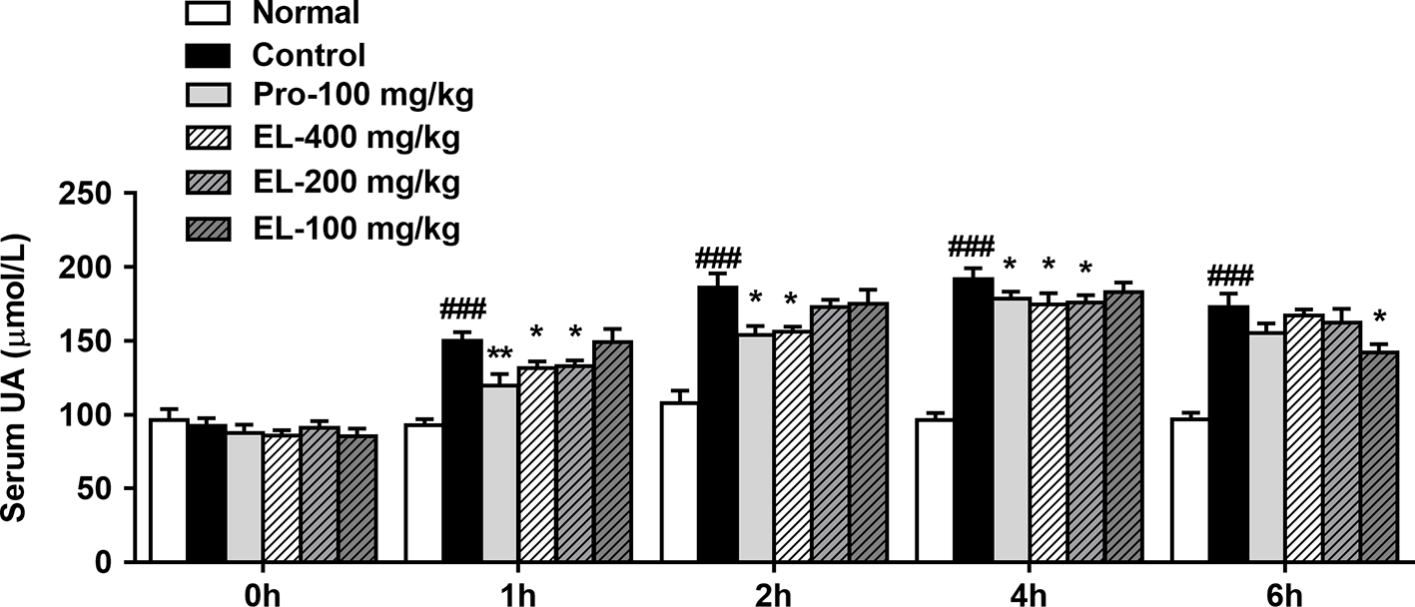
Effects of EL on the levels of serum uric acid in hyperuricemic rats. Normal, normal control group; Control, PO induced hyperuricemia group; Pro-100, positive control probenecid group; EL, *E. longifolia* stem extract in different dosage group. Values represent the mean ± S.E.M. of determinations; *P < 0.05, **P < 0.01, vs. hyperuricemia group, ^###^P < 0.001 vs. normal control group.

As shown in **Figure 2**, oral administration of PO could significantly elevate the rat serum uric acid level in 1–6 h, indicating that the model was established successfully. Compared to the hyperuricemia control group, the serum uric acid of the positive control (100 mg/kg probenecid) group was significantly decreased in the 1–4 h. Similarly, 400 mg/kg EL treatment remarkably reduced serum uric acid levels in 1–4 h, at 6 h, 100 mg/kg EL significantly reduced blood uric acid levels (*p* < 0.05) (**Figure 2**).

### EL Reduced Plasma Uric Acid Levels and Enhanced Uric Acid Excretion in PO and Adenine Induced Hyperuricemia Mice

**Figure 3:**
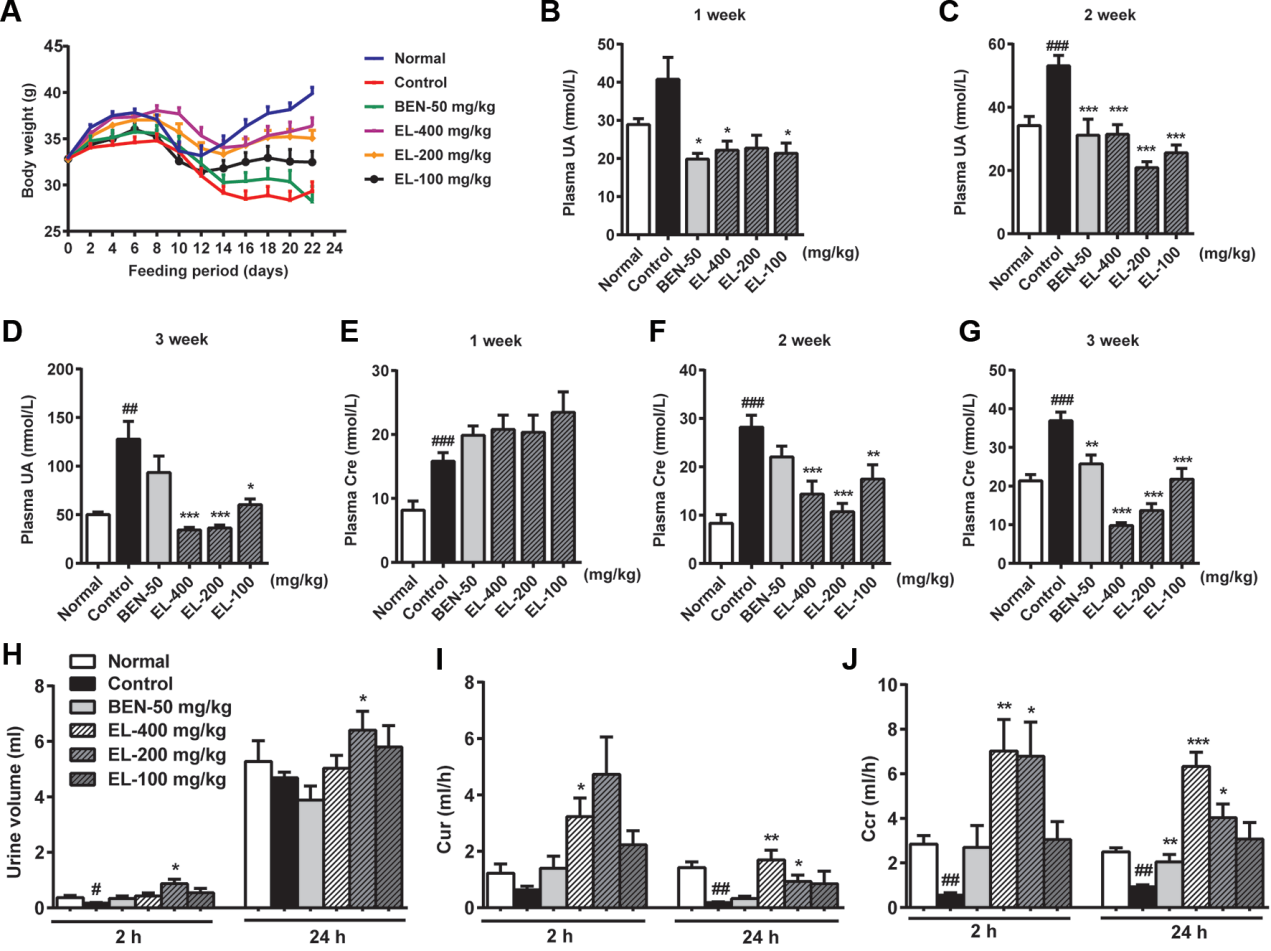
EL reduced plasma urate levels and enhanced excretion of urate in adenine and PO induced hyperuricemia mice. **(A)** Body weight changing curve; **(B**–**D)** plasma uric acid levels at 1, 2, 3 weeks after administration; **(E**–**G)** plasma creatinine levels at 1, 2, 3 weeks after administration; **(H)** Urine volume; **(I)** 2 h and 24 h Cur; **(J)** 2 h and 24 h Ccr. Normal, normal control group; Control, PO induced hyperuricemia group; Ben-50, positive control benzbromarone group; EL, *E. longifolia* stem extract in different dosage group. Values represent the mean ± S.E.M. of determinations (n = 8–10); *P < 0.05, **P < 0.01, ***P < 0.001 vs. hyperuricemia group, ^#^P < 0.05, ^##^P < 0.01, ^###^P < 0.001 vs. normal control group.

As shown in **Figure 3A**, the mice body weight growths were recorded, and the control group mice showed a significant reduction in body weight compared to the normal group. Compared with the control group, the mice body weight was significantly increased in 400 and 200 mg/kg EL groups. While there was no significant difference between benzbromarone group and control group mice.

Plasma uric acid levels were much higher in the PO induced control group than normal group at 2–3weeks (*p* < 0.01). Oral administration of EL at 100, 200, and 400 mg/kg could markedly decrease plasma uric acid levels in hyperuricemia mice at 2–3 weeks, indicating the anti-hyperuricemia effects of EL. In addition, benzbromarone (50 mg/kg) as a positive control also significantly reduced plasma uric acid levels in hyperuricemia mice at 1–2 weeks (*p* < 0.05) (**Figures 3B–D**).

To determine renal function, creatinine level was examined. Plasma creatinine levels were significantly higher in the hyperuricemia group than normal control group (*p* < 0.001) Benzbromarone significantly reduced plasma creatinine levels at 3 weeks (*p* < 0.01). EL at 100, 200, and 400 mg/kg also significantly reduced plasma creatinine levels in Hyperuricemia mice at 2–3 weeks (*p* < 0.01) (**Figures 3E–G**).

To evaluate the effect of EL on uric acid excretion, Cur and Ccr were calculated. In the hyperuricemia group, 24 h Cur and Ccr were significantly decreased compared with normal mice (*p* < 0.01), while Cur and Ccr were recovered in hyperuricemia mice treated with 200 and 400 mg/kg EL (*p* < 0.01) (**Figures 3H–J**).

### EL Ameliorated Kidney Histological Alterations in PO and Adenine Induced Hyperuricemia Mice

**Figure 4:**
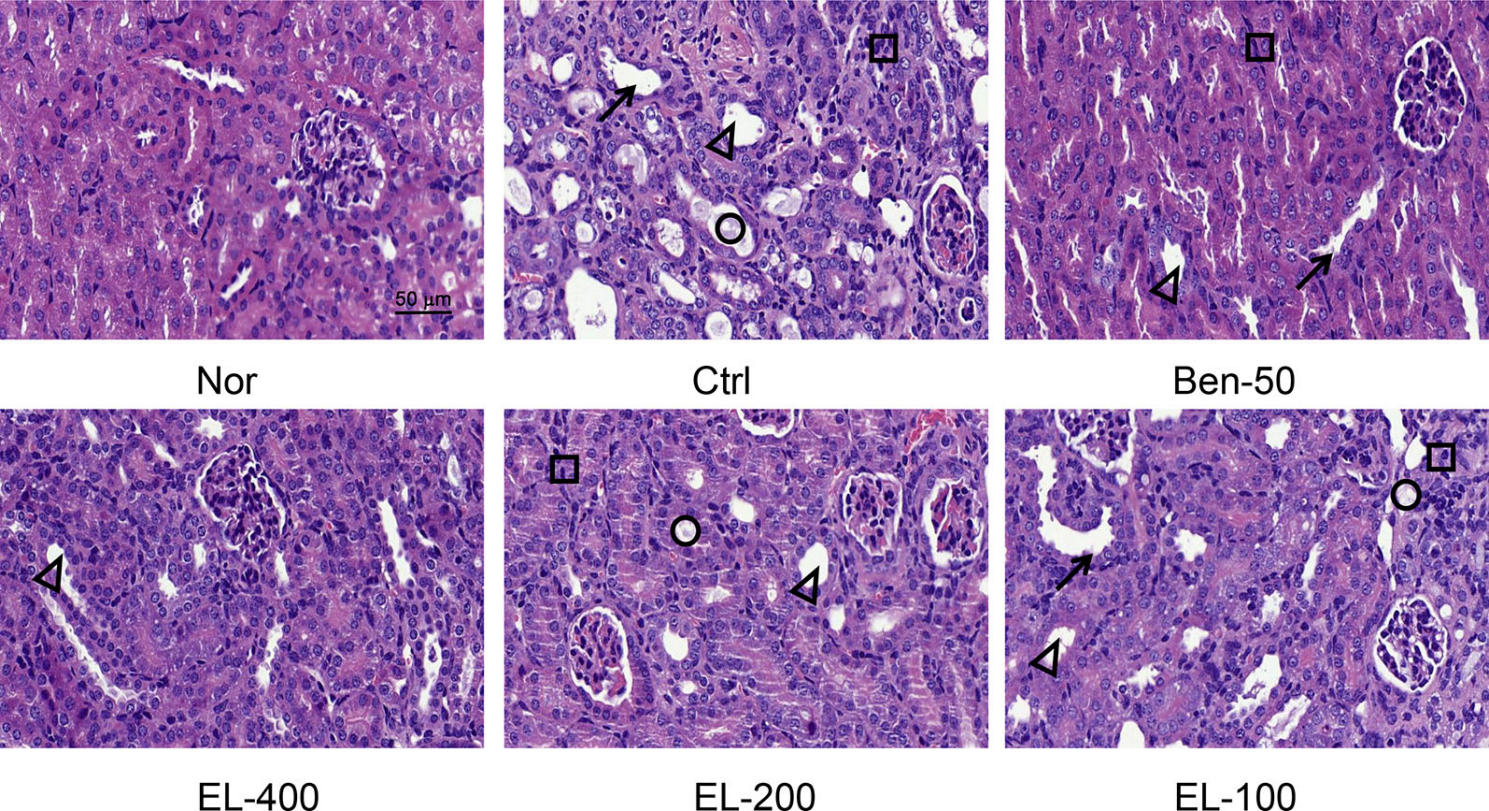
EL improved kidney histological alterations in adenine and PO induced mice. Kidney sections for H&E staining (400x). Nor, normal control group; Ctrl, adenine and PO induced hyperuricemia group; Ben-50, positive control benzbromarone group; EL, *E. longifolia* stem extract in different dosage group. Black arrow, necrotic tubular epithelial, Black triangle, tubular ectasia, Black circle, corpora amylacea, Black square, cellular infiltration

As shown in **Figure 4**, kidney tissue sections were stained with H&E. In the normal group, the boundary of the renal tubules was clear and the epithelial cells were arranged neatly. PO and adenine induced hyperuricemia mice exhibited severe renal tubule dilation, mild inflammatory cell infiltration, inconspicuous of boundaries between adjacent proximal tubule cells, renal tubules with protein casts, swelling, and proximal tubule necrosis. Benzbromarone and ELs treatments significantly alleviated the hyperuricemia induced renal damage. EL at high dose exhibited a better effect than low dose.

### EL Modulated Renal Urate Transport-Associated Proteins in PO and Adenine Induced Hyperuricemic Mice Kidneys

**Figure 5:**
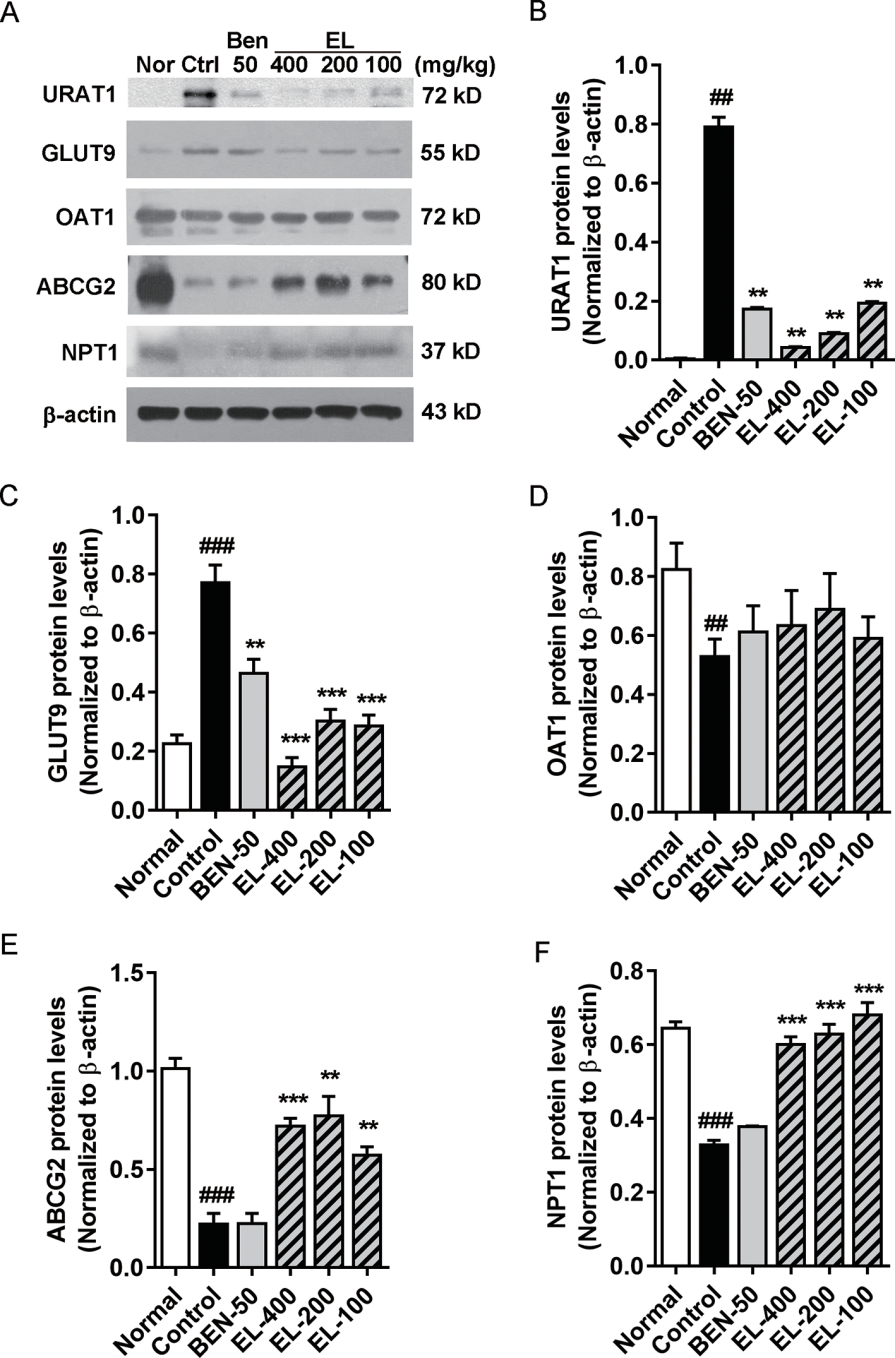
EL modulates renal urate transport-associated protein expression levels in kidneys of adenine and PO induced hyperuricemic mice **(A)** Expression of URAT1, GLUT9, OAT1, ABCG2, and NPT1 in mice kidney. **(B**–**F)** The Western blot analysis of URAT1, GLUT9, OAT1, ABCG2, and NPT1 relative protein expression levels. Values represent the mean ± S.E.M. of determinations; **P < 0.01, ***P < 0.001 vs. hyperuricemia group, ^##^P < 0.01, ^###^P < 0.001 vs. normal control group.

The effects of EL on renal URAT1, GLUT9, ABCG2, OAT1, and NPT1 protein expression were detected (**Figure 5**). Compared to the normal group, the protein expression levels of GLUT9 and URAT1 (*p* < 0.01) were significantly induced in hyperuricemia mice. By contrast, benzbromarone and EL treatment significantly reduced the expression levels of URAT1 and GLUT9 (p < 0.01). In addition, compared with the normal group, the ABCG2, OAT1, and NPT1 protein expressions were significantly decreased in the hyperuricemia control group (p < 0.01). EL treatment increased NPT1 and ABCG2 protein expression levels (p < 0.01), but it had no effect on OAT1 expression.

### Inhibitory Effects of Constituents in EL on Urate Uptake in hURAT1-Expressing HEK293T Cells

**Figure 6:**
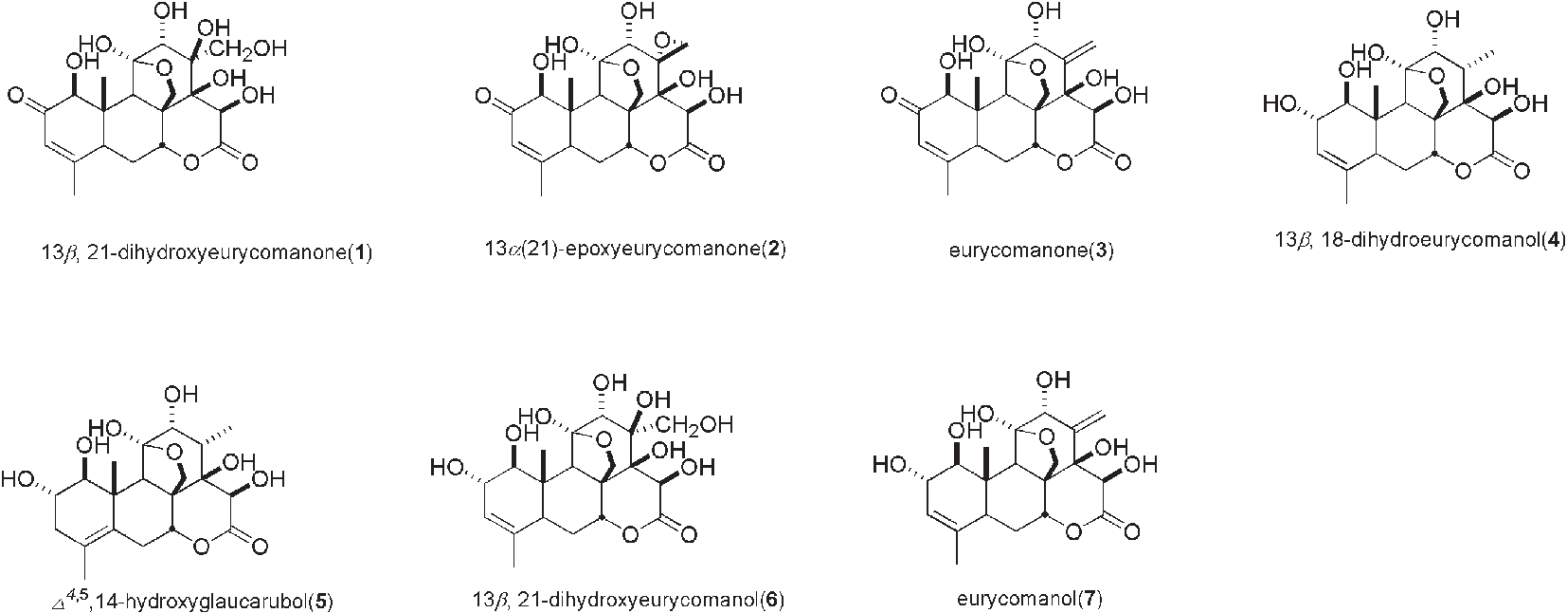
The structure of compounds **1**–**7**.

**Figure 7:**
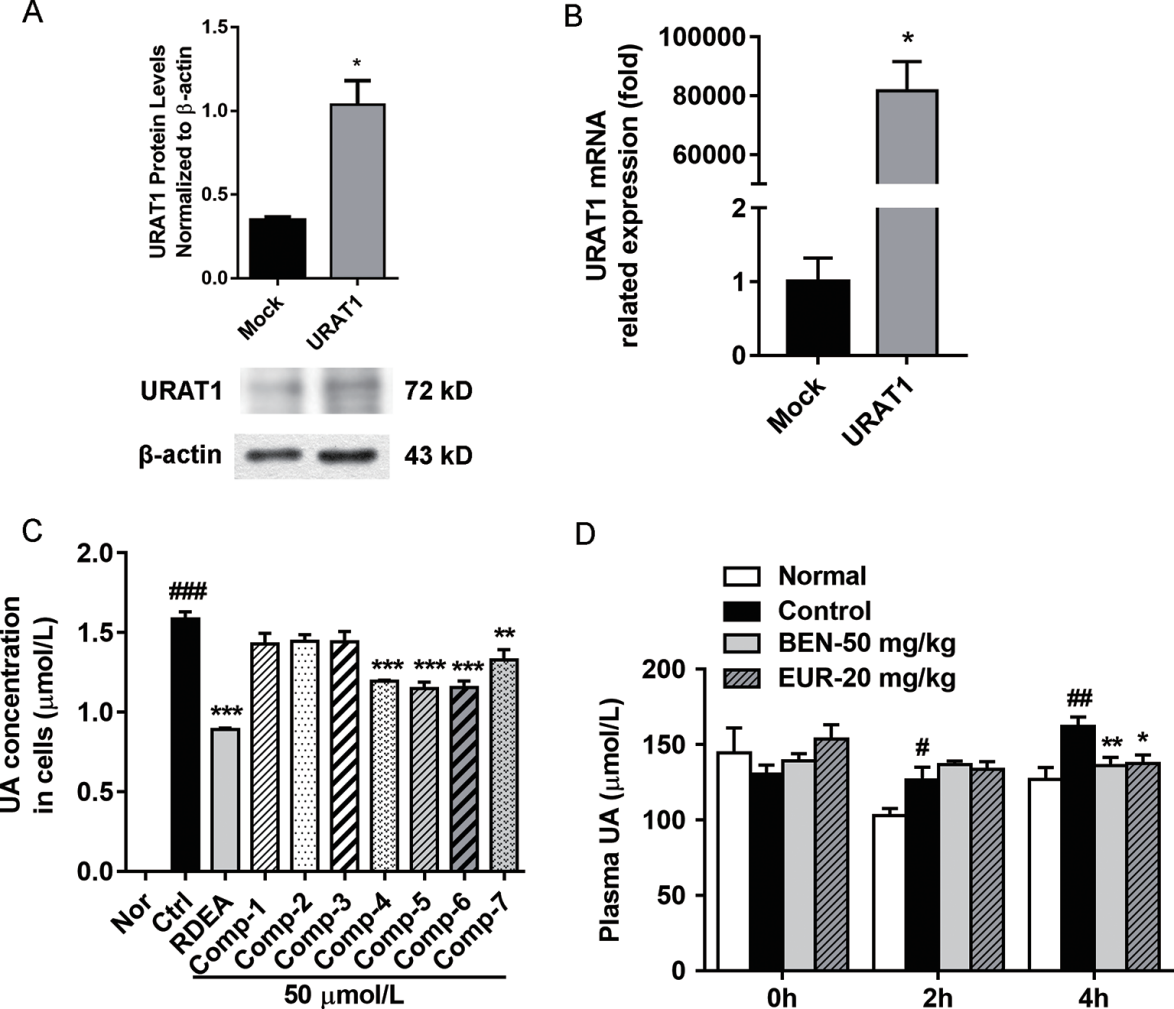
Eurycomanol treatment reduced the uric acid levels *in vitro* and *in vivo*. **(A)** The protein levels of URAT1 in hURAT1-expressing HEK293 cells. **(B)** The mRNA levels of URAT1 in hURAT1-expressing HEK293 cells. **(C)** Effects of constituents on uric acid uptake in hURAT1-expressing HEK293 cells. **(D)** Plasma uric acid levels in hyperuricemic mice. Values represent the mean ± S.E.M. of determinations; *P < 0.05, **P < 0.01, ***P < 0.001 vs. hyperuricemia group, ^#^P < 0.05, ^##^P < 0.01, ^###^P < 0.001 vs. normal control group.

As shown in **Figure 6**, we identified seven major quassinoids from EL. The isolation procedure was shown as supplement 1. By means of comparing their chemical and spectroscopic data with those reported in literatures, the structures were identified as 13β, 21-dihydroxyeurycomanone (**1**) (Morita et al., 1990), 13α(21)-epoxyeurycomanone (**2**) (Morita et al., 1993), eurycomanone (**3**) (Morita et al., 1990), 13β, 18-dihydroeurycomanol (**4**) (Chan et al., 1991), Δ^4,5^,14-hydroxyglaucarubol (**5**) (Meng et al., 2014), 13β, 21-dihydroxyeurycomanol (**6**) (Kuo et al., 2004), and eurycomanol (**7**) (Chan et al., 1991). Since URAT1 plays a key role in urate reabsorption in kidney, hURAT1-expressing HEK293T cells were used to determine the effects of urate uptake regulation role of quassinoids components from EL. The URAT1 protein and mRNA expression levels in hURAT1-expressing HEK293T cells were significantly increased compared to empty vector-transfected HEK293T cells (p < 0.05) (**Figures 7A, B**). As shown in **Figure 7C**, urate absorption was significantly increased in hURAT1-expressing cells. RDEA3170 (1 µmol/L) and compound **4**–**7** (50 µmol/L) treatment could decrease the urate uptake in hURAT1-expressing cells (p < 0.01), whereas compound **1**–**3** showed comparatively low activities, which indicated that the URAT1 inhibitory effect of EL could result from the activity of eurycomanol type quassinoids.

### Eurycomanol Inhibited Plasma Uric Acid Levels Increasing in PO Induced Hyperuricemic Mice

Further, we tested the *in vivo* activity of eurycomanol, which showed better activity on URAT1 inhibition. As shown in **Figure 7D**, compared to the normal group, oral administration of PO could significantly elevate the plasma uric acid in 2–4 h (*p* < 0.05). However, eurycomanol significantly reduced plasma uric acid levels at doses of 20 mg/kg in 4 h (*p* < 0.05). These results further confirm that eurycomanol type quassinoids were the active compounds in EL on hyperuricemia.

## Discussion

Hyperuricemia is caused either due to excessive production or insufficient excretion of uric acid. Notably, current literature indicate that about 60% of hyperuricemia with lower urinary urate excretion states (Campo et al., 2003). Uric acid handling by kidney can be divided into four processes, involving glomerular filtration, tubular reabsorption and secretion, and post-secretory reabsorption. This process is mediated by renal transporters (reabsorptive and secretory transporters) in the proximal tubule of the kidney (Sakhaee et al., 2002), including re-absorptive urate exchangers URAT1, organic anion transporter 4, 10 (OAT4 and OAT10), GLUT9, and secretory exchange transporters ABCG2, multidrug resistance-associated protein (MRP4), NPT1, and NPT4. The plasma urate can be almost freely filtered by the glomerulus to the renal tubules, and then 90% of the filtered urate is reabsorbed back into blood (Hyndman et al., 2016). Among these, URAT1 and GLUT9 contribute most on the reabsorption of uric acid, are the main aiming targets of anti-hyperuricemia during the new drug development.

As a potential URAT1 inhibitor, lesinurad was developed by AstraZeneca in 2012. In vitro research revealed that lesinurad significantly affected URAT1 with IC_50_ of 7.18 µmol/L (Cai et al., 2018), but clinic trials were not optimistic. With lesinurad monotherapy, patients did not achieve individualized serum urate target of <5 mg/dL, serum creatinine increased of unknown origin, and renal side effects occurred in a significant percentage and thus prompted AstraZeneca to stop for market authorization of monotherapy lesinurad 400 mg (Jansen et al., 2018).

The above evidence suggested that a selective inhibitor monotherapy is difficult to achieve the desired therapeutic target. Uric acid excretion is a complex transport system involving multifactorial of molecular events, and integrative multi-target drug intervention is considered more efficient in modulating networks than targeting a single macromolecule with a high-affinity ligand.

EL is a traditional herbal medicine used in southeast Asia. The alcohol or hot water extract was used for health care, such as aches, persistent fever, malaria, and sexual insufficiency (Bhat and Karim, 2010). In this study, we found that EL significantly decreased serum uric acid levels and increased Cur and Ccr. Previously, our in-house data showed that EL and its major compound eurycomanol and eurycomanone have no significant inhibitory activities on XOD, which indicated that the effect of EL on hyperuricemia is not due to inhibition of uric acid production. We focused our attention on uric acid excretion.

Subsequently, protein analysis revealed that oral administration of EL remarkably decreased renal URAT1 (0.78 folder vs. control) and GLUT9 (0.81 folder vs. control) expression levels. Increased renal NPT1 (1.1 folder vs. control) as well as ABCG2 (2.5 folder vs. control) expressions. These results indicated that EL enhanced uric acid excretion and reduced its re-absorption.

URAT1 is a key carrier for uric acid re-absorption. Genotype analysis revealed that mutations in the URAT1 and GLUT9 genes are a causative factor of renal hypouricemia linked to hyperuricemia and gout (Vazquez-Mellado et al., 2007; Matsuo et al., 2008). In renal urate-handling processes, firstly, as a key UA carrier, URAT1 transfers UA from basolateral membrane to proximal tubular cell, and then, GLUT9 is responsible for exchange urate from the cell to the peritubular interstitium (Atsushi et al., 2002). In hyperuricemia patient, URAT1 activity is significantly increased and more responsive renal re-absorption of uric acid (Tan et al., 2016).

In present study, we found that EL significantly reduced serum UA level and significantly decreased renal URAT1 and GLUT9 expressions. We hypothesize that this effect may be due to reduce of URAT1 activity. As a further study, seven compounds of EL were screened using a cell model with overexpressed URAT1. Among them, quassinoids exhibits inhibition of urate uptake *in vitro*, suggesting that quassinoids may be the main active components of EL. Subsequently, we confirmed the activity of eurycomanol *in vivo*, which reduced serum uric acid level in PO induced hyperuricemia mice.

As a summary, our findings revealed that EL significantly reduced blood uric acid levels and prevented pathological changes of kidney in PO induced hyperuricemia animal model, improved renal urate transports. We partly clarified the mechanism was related to URAT1 inhibition by quassinoids in EL. EL and eurycomanol are useful substances for prevention or treatment on hyperuricemia through increasing uric acid excretion.

## Data Availability Statement

All datasets generated for this study are included in the article/**Supplementary Material**.

## Ethics Statement

The animal study was reviewed and approved by Science and Technological Committee and the Animal Use and Care Committee of TJUTCM.

## Author Contributions

RB, ML, YZha, and TW contributed to experimental design. RB, DW, SW, HY and YZho contributed to the acquisition and analysis of data. ML, DW and ZL reviewed the manuscript. TW obtained the funding. RB and TW wrote the manuscript.

## Funding

This research was supported by Important Drug Development Fund, Ministry of Science and Technology of China (2018ZX09735-002) and National Natural Science Foundation of China (81173524; 81673688).

## Conflict of Interest

Yi Zhong was employed by Global Education Network Sdn.Bhd.

The remaining authors declare that the research was conducted in the absence of any commercial or financial relationships that could be construed as a potential conflict of interest.

## Abbreviations

EL, *E. longifolia* stem extract; PO, Potassium oxonate; UPLC, Ultra Performance Liquid Chromatography; H&E, Hematoxylin-eosin staining; URAT1, urate reabsorption transporter 1; GLUT9, Glucose transporter 9; ABCG2, ATP-binding cassette transporter G2; XOD, Xanthine oxidase; OAT1-3, Organic anion transporter 1-3; OAT4, Organic anion transporter 4; OAT10, Organic anion transporter 10; NPT1, Sodium-dependent phosphate transporter 1; NPT4, Sodium-dependent phosphate transporter 4; MRP4, Multidrug resistance-associated protein; HPLC, High-performance liquid chromatography; Cur, Clearance of uric acid; Ccr, Clearance of creatinine; TBST, Tris Buffered Saline with Tween 20.
