# Supplementary material for: Effect of Eurycoma longifolia Stem Extract on Uric Acid Excretion in Hyperuricemia Mice
Source: Front Pharmacol. 2019 Dec 10;10:1464. doi: 10.3389/fphar.2019.01464 (PMC6914847; doi:10.3389/fphar.2019.01464)
Supplement: Supplementary file 1 [file DataSheet_1.docx]

**Supplement 1. Isolation procedure of diterpenes from stems of Eurycoma longifolia**

**General**

NMR spectra were run on a Bruker 500 MR NMR spectrometer (Bruker BioSpin AG Industriestrasse 26 CH-8117, Fällanden, Switzerland) at 500 MHz for ^1^H and 125 MHz for ^13^C NMR (internal standard: TMS). Positive- and Negative-ion mode HRESI-TOF-MS were measured on an Agilent Technologies 6520 Accurate-Mass Q-Tof LC/MS spectrometer (Agilent Corp., Santa Clara, CA, USA). [α]_D_ spectrum was determined on a Rudolph Autopol^®^ IV automatic polarimeter (l = 50 mm) (Rudolph Research Analytical, Hackettstown NJ, USA).

Column chromatography (CC) were performed over macroporous resin D101 (Haiguang Chemical Co., Ltd., Tianjin, China), ODS (50 μm, YMC Co., Ltd., Tokyo, Japan). Preparative high-performance liquid chromatography (pHPLC) column: Venusil PrepG C18 (50 mm i.d. × 250 mm, 10 µm, Agela technologies, Tianjin, China) and Cosmosil PBr (20 mm i.d. × 250 mm, 5 µm, Nacalai Tesque, Inc., Kyoto, Japan); and were used to separate the constituents.

**Plant Material**

The stems *Eurycoma longifolia* Jack were collected from Nuang Mountain Recreation Forest in Selangor city, Malaysia, and identified by Dr. Wang Tao (Institute of Traditional Chinese Medicine, Tianjin University of Traditional Chinese Medicine).

**Extraction and Isolation**

The dried stems of *E. longifolia* (3.4 kg) were cut to pieces and refluxed with 70% EtOH to gain 70% EtOH extract (EL) 160.0 g (4.7%). Then, EL (125.0 g) was partitioned in an EtOAc-H_2_O mixture (1:1, v/v) to obtain EtOAc layer (35.5 g, 1.3%) and H_2_O layer (85.0 g, 3.2%), respectively. The H_2_O layer was subjected to D101 macroporous resin CC (H_2_O → 95% EtOH). And H_2_O (56.8 g, 2.1%) and 95% EtOH (31.3 g, 1.2%) eluates were obtained.

95% EtOH eluates (25.0 g) was subjected to ODS CC [MeOH-H_2_O (10:90 → 20:80 → 30:70 → 40:60 → 50:50→ 60:40 → 100:0, v/v)], and 19 fractions (ELG1–ELG19) were yielded. ELG5 (875.5 mg) was separated by pHPLC [CH_3_CN-1% HAc (12:88, v/v), Cosmosil PBr column] to obtain 13*β*,21-dihydroxyeurycomanone (**1**, 73.9 mg) and 13*α*(2l)-epoxyeurycomanone (**2**, 117.2 mg). ELG6 (1570.0 mg) was isolated by pHPLC [CH_3_CN-1% HAc (14:86, v/v), Cosmosil PBr column] to produce eurycomanone (**3**, 655.0 mg). ELG10 (2170.0 mg) was subjected to pHPLC [CH_3_CN-1% Hac (9:91, v/v), Cosmosil PBr column], and 7 fractions (ELG10-1–ELG10-7) were given. ELG10-1 (90.6 mg) was further purified by pHPLC [CH_3_CN-1% HAc (12:88, v/v), Cosmosil PBr column] to yield 13*β*,21-dihydroxyeurycomanol (**6**, 25.2 mg). ELG11 (2.1 g) was subjected to pHPLC [CH_3_CN-1% Hac (12:88, v/v), Venusil PrepG C18] to gain nine fractions (ELG11-1–ELG11-9). Among them, fraction 11-6 was identified as eurycomanol (**7**, 1040.0 mg). ELG11-7 (175.8 mg) was purified by pHPLC [CH_3_CN-1% Hac (16:84, v/v), Cosmosil PBr column] to give 13*β*,18-dihydroeurycomanol (**4**, 9.7 mg) and *Δ*^4,5^,14-Hydroxyglaucarubol (**5**, 6.9 mg).

MTT viability assay
